# Chronic Chest Pain Control after Trans-Thoracic Biopsy in Mediastinal Lymphomas

**DOI:** 10.3390/healthcare9050589

**Published:** 2021-05-18

**Authors:** Antonello Sica, Beniamino Casale, Caterina Sagnelli, Maria Teresa Di Dato, Marco Rispoli, Mario Santagata, Pietro Buonavolontà, Alfonso Fiorelli, Paola Vitiello, Stefano Caccavale, Massimiliano Creta, Anna Maria Salzano, Evangelista Sagnelli, Elisabetta Saracco, Giuseppe Gazzerro, Vincenzo Famiglietti, Dario Tammaro, Alfonso Papa

**Affiliations:** 1 Department of Precision Medicine, University of Campania Luigi Vanvitelli, 80131 Naples, Italy; vincenzo.famiglietti@yahoo.it; 2 Department of Pneumology and Tisiology, AORN Dei Colli-V. Monaldi, 80131 Naples, Italy; benny.casale@hotmail.com; 3 Department of Mental Health and Public Medicine, University of Campania Luigi Vanvitelli, 80131 Naples, Italy; caterina.sagnelli@unicampania.it (C.S.); evangelista.sagnelli@unicampania.it (E.S.); 4 Pain Department, AORN Dei Colli-V. Monaldi, 80131 Naples, Italy; marisita@libero.it (M.T.D.D.); marco-rispoli@hotmail.it (M.R.); pietrobuonavolonta@libero.it (P.B.); annamaria.salzano@ospedalideicolli.it (A.M.S.); elisabetta.saracco@ospedalideicolli.it (E.S.); gazzerro.aaroicolli@gmail.com (G.G.); dariotammaro@libero.it (D.T.); alfonsopapa@libero.it (A.P.); 5 Multidisciplinary Department of Medical Surgery and Dental Specialties, University of Campania Luigi Vanvitelli, 80131 Naples, Italy; mario.santagata@unicampania.it; 6 Thoracic Surgery Unit, University of Campania Luigi Vanvitelli, 80131 Naples, Italy; alfonso.fiorelli@unicampania.it; 7 Dermatology Unit, University of Campania, 80131 Naples, Italy; paoladermosun@libero.it (P.V.); stefano85med@gmail.com (S.C.); 8 Department of Advanced Biomedical Sciences, University of Naples Federico II, 80131 Naples, Italy; Massimiliano.creta@unina.it

**Keywords:** chronic pain, mediastinal j, trans-thoracic biopsy

## Abstract

Chest pain following a trans-thoracic biopsy often has multiple etiologies, especially in patients with lymphomas. Pathological neuronal mechanisms integrate with an overproduction of IL-6, TNF-α, IL1-β by macrophages and monocytes, which amplifies inflammation and pain. In consideration of this complex pathogenesis, international guidelines recommend diversified analgesia protocols: thoracic epidural, paravertebral block, and systemic administration of opioids. This study reports an attempt to reduce chest pain and prevent chronic pain in 51 patients undergoing trans-thoracic biopsy for mediastinal lymphoma. The entity of pain, measured 72nd hour after biopsy by the Numerical Rating Scale (NRS), was compared with that seen at a 6th month checkpoint in 46 patients. The pain decreased in all cases. At the 6th month checkpoint, among 31 opioid-treated patients, none of the 16 patients with NRS < 6 within the 72nd hour post biopsy had developed chronic chest pain, while 8 of the 15 with higher values did (*p* < 0.01). Of 10 patients undergoing thoracotomy and treated with opioids, eight had a NRS of no more than 2, of which six had no chronic pain. Of the twenty-one patients who underwent VATS biopsy and were treated with opioids, fifteen had NRS no greater than 2, of which ten had no chronic pain. Subgroups of patients biopsied under mediastinotomy or video-assisted thoracoscopic surgery (VATS) and treated with thoracic epidural analgesia (TEA) or PVB were too small for such analysis.

## 1. Introduction

Chest pain caused by a trans-thoracic biopsy is a complex pathophysiological entity, not exclusively dependent on trauma, but often caused by various etiological factors, especially in elderly patients with lymphomas or other neoplasms. Prolonged post-traumatic inflammation can induce persistent stimulation of the nociceptive pathways beyond recovery, thus generating chronic pain [1,2,3,4,5]. The neuronal pathophysiological mechanisms integrate with an immunological response [6,7,8], characterized by an overproduction of cytokines such as IL-6, TNF-α, IL1-β by macrophages and monocytes, with amplification of inflammation and pain. Both B lymphocytes and activated T lymphocytes have an inhibitory action on pain through the production of IL-10, the cytokine with the highest inhibitory action on the secretion of IL-1β, IL-6 and TNF-α [9,10,11,12,13,14,15]. Especially in lymphomas, but also in other neoplasms, the physio-pathological mechanisms involving these cells are altered and this helps to contain the amplification of neuropathic pain [16,17,18,19,20,21,22,23].

Thoracic incision is among the most painful types of incisions a patient can undergo [24], due to an intercostal nerve injury related to the spread of the ribs. This pain has the typical pattern of nociceptive pain with a neuropathic component: intense, long-lasting, severe, up to 72–96 h and with a tendency to chronicity. Pain can inhibit effective coughing and deep breathing; consequently, pulmonary ventilation is reduced, with an increased risk of lung infections, especially in the elderly [25,26]. Furthermore, inadequate management of post-operative pain may contribute to the development of a post-thoracotomy chronic pain syndrome [27,28,29,30,31,32,33,34,35]. The complexity of the pathogenesis of pain due to trans-thoracic biopsy does not foresee univocal solutions, other than an early mobilization after surgery.

Thoracic epidural analgesia (TEA) is commonly considered the gold standard for limiting post-operative pain, as it has been shown to reduce the risk of persistent post-operative pain (PPP) more effectively than other measures; in some cases, however, it is contraindicated and in others it may not work [36]. In addition, some patients undergoing epidural analgesia may develop some adverse reactions, such as hypotension, epidural hematoma, nerve damage and shoulder pain on the ipsilateral side of the incision [37,38,39,40].

The origin of this pain has not been fully understood and alternative methods for postoperative pain control are considered of enormous interest [41,42]. Postoperative neuropathic pain may be inevitable for some high-risk patients. Several factors are responsible for the development of chronic pain after thoracotomy such as damage of the intercostal nerves, the location and duration of surgery, the age of patients, history of smoking, preoperative use of hypnotic drugs, primary lung cancer, pneumothorax, malignant diseases, partial resection, segmentectomy, complete VATS, intraoperative blood loss, and duration of the chest tube. In particular, the preoperative use of hypnotic drugs and the long duration of surgery (≥2.5 h) were identified as risk factors for post-operative neuropathic pain in patients who did not receive epidural analgesia, in those with impaired renal function, and in those undergoing a comprehensive VATS approach. The psychosocial condition of patients, including anxiety and depression due to the severity of the illness, the lack of a social support network, a poor social status, and the fear of the acute and chronic postoperative pain, affects the perception and the effects of chronic neuropathic pain regardless of the surgical approach or pain management techniques. Long duration of surgery (≥2.5 h) is another risk factor of neuropathic pain. Thoracic surgery routinely crushes the intercostal nerves, particularly where the nerves are exposed along the caudal side of the rib. Intercostal nerve damage might increase proportionately with the duration of surgery, but the complete VATS approach could minimize intercostal nerve damage.

Systemic opioid administration is the simplest and most common method of providing analgesia for postoperative pain, but this can be associated with several side effects, such as respiratory depression, sedation, nausea, and vomiting. The guidelines of the American Pain Society of Regional Anesthesia recommends multimodal analgesia protocols. In this regard, the Italian Society of Anesthesia Analgesia Resuscitation, and Intensive Care (SIAARTI) has adopted multimodal strategies since 2010 and has stressed the need to combine techniques of systemic and local anesthesia, and to reduce the use of opiates, a suggestion reaffirmed in 2019. Today we act at various levels, periphery, nociceptors, and afferent conduction pathways, trying to optimize the advantages of each drug and limit its side effects. The treatment begins at the time of the surgical incision, acting early on the nociceptive afferents, and then continues over time. The Cochrane meta-analysis of 14 studies with 698 patients undergoing thoracotomy, compared paravertebral block (PVB) with TEA and found no difference in the 30-day post-operative mortality rate, in the incidence of cases with excessive sedation and in the duration of the hospital stay; in terms of analgesic efficacy, PVB was comparable to TEA, but had a lower incidence of cases with hypotension, nausea, vomiting, itching, and urinary retention [43,44,45,46,47].

The present study reports our attempts to reduce chest pain and prevent chronic pain in 51 patients undergoing transthoracic biopsy for suspected mediastinal lymphoma, with an open approach or with a minimally invasive approach such as video-assisted thoracoscopic surgery (VATS). The aim of this study was to highlight which of the surgical techniques and anesthetic choices (systemic opioid analgesia, TEA or PVB) [48,49] used in the study proved more effective in reducing post-operative pain. We also paid particular attention to verifying whether the intensity of chest pain detected within the 72nd hour after surgery could have a predictive value for the development of chronic pain.

## 2. Materials and Methods

### 2.1. Patients

Fifty-one consecutive patients undergoing transthoracic biopsy for suspected mediastinal lymphoma from April 2018 to October 2019 at the Department of Pain, AORN Dei Colli, Naples, Italy, were enrolled in this study. Needle biopsy is a common procedure for diagnosing anterior mediastinal tumors, but in some cases, only necrotic or fibrotic material is recovered and sometimes tumor samples are too small to allow diagnosis and a complete immunohistochemical typing. The present study includes only patients with mediastinal lymphoma. The age of the patients ranged from 57 to 84 years (median 68); 31 were males and 20 females. No patient had suffered from chronic chest pain or had undergone systemic analgesic therapy prior to trans-thoracic biopsy. Patients were followed by hospital doctors during hospitalization and entrusted to their family doctor after discharge with clear written instructions on how to continue the clinical follow-up and how to practice any occasional analgesic therapies. If deemed necessary by the family doctors, the patients were re-evaluated by the hospital doctors and then returned to the family doctor with clear written instructions on how to continue the clinical follow-up. Six months after the trans-thoracic biopsy, 46 of the 51 enrolled patients were re-evaluated by hospital physicians as outpatients.

The thoracic pain was evaluated with the McGill Pain Questionnaire (MPQ) [50], recording the Pain Rating Index (PRI) (range 0 no pain−78 worst pain), and with the Numerical Rating Scale (NRS) (scale 0–10: 0 = no pain, 10 = worst pain ever), within the 72nd hour after surgery and 6 months later; particular attention was paid to evaluate whether the score detected within the 72nd hour after biopsy might have a predictive value of chronic chest pain development. Changes in discomfort and quality of life was assessed by the Brief Pain Inventory Short Form.

Patients with lymphoma were treated with chemotherapy after the histological diagnosis was made, in accordance with international guidelines. No patient underwent radiotherapy in the six months following surgery, as it was not necessary at that stage.

### 2.2. Ethics Approval

All clinical procedures were performed in accordance with international guidelines and with the Helsinki Declaration of 1975, revised in 1983. The study was approved by the local Ethics Committees named: “Comitato Etico Universita’ Degli Studi Della Campania “Luigi Vanvitelli”-Azienda Ospedaliera Universitaria “Luigi Vanvitelli”-Azienda Ospedaliera Rilievo Nazionale “Ospedali Dei Colli”, Naples, Italy, with protocol number 263/2018.At the baseline visit, each patient signed an informed consent for the use of their data in clinical investigation, in keeping with the Italian lows on privacy.

### 2.3. Surgical Techniques

The surgical techniques for performing biopsy were open biopsy via anterior mediastinotomy (also known as Chamberlain’s procedure) or VATS biopsy.

Anterior Mediastinotomy: the procedure was performed in an operating room under general anesthesia and endotracheal intubation. The patient was placed in a supine position and a 4 cm incision was performed in the parasternal 2nd intercostal space. The cartilage of the third rib was removed with the preservation of the perichondrium and the mammary artery. Rib retractor was used to magnify surgical filed. The extra-pleural space was opened by blunt dissection to push the pleura away from the mediastinum and to gain access to the target lesion. Biopsies of the lesion were performed with standard biopsy forceps and tissue was evaluated for adequacy. At the end of the procedure, the cartilage was replaced, and the wound closed in layers. No chest drainage was placed.

VATS biopsy: The procedure was performed with a single incision in the operating room, under general anesthesia and with selective endotracheal intubation. A 2–3 cm incision was made within the 4th intercostal space on the anterior axillary line and protected by a wound retractor. No rib retractor was used. This access was used for work tools and the camera. The lung was gently retracted to access the anterior mediastinum, and the target lesion was biopsied. The tissue samples thus obtained were evaluated for adequacy. At the end of the procedure a 24 French chest tube was inserted through the same incision and removed the next day.

### 2.4. Anesthetic Techniques

The anesthetic techniques used were TEA, PVB, and opioid analgesia.

For TEA, patients were placed in a sitting position with the neck and upper back in flexion; the approach to the thoracic epidural space was performed using as superficial anatomical reference points to approximate the puncture site to the intended segment, the prominent spinous process of C7 and the lower edge of the scapula (T7). Skin anesthesia was performed with lidocaine 2% 3 mL; the 18 G Tuohy needle was advanced with the loss of resistance technique by means of an “air” syringe. Once the epidural space was found, ropivacaine 0.5% 6 mL was administered, and a catheter was placed and then fixed to the skin and connected to an elastomer for continuous administration of local anesthetic. The whole method was performed in a sterile condition. Four hours after the epidural anesthesia, ropivacaine 0.2% at 5 mL/h was administered intravenously for 72 h.

For PVB, the injection of local anesthetic was administered at the T3 level with the patient in lateral position with the site of surgery on the top. The ultrasound probe was placed 5–6 cm from the midline in the craniocaudal direction and moved medially to identify the transverse process and parietal pleura. Eight ml of ropivacaine 0.5% were deposited in the space between the pleura and the costotransverse ligament by a 100 mm needle positioned out of plain. The same procedure was performed at the T4 and T5 level homolaterally. A catheter was placed at the T 4 level and connected to an elastomer for continuous administration of local anesthetic. The whole method was performed in a sterile condition. Four hours after blockade, ropivacaine 0.2% at 12 mL/h was administered intravenously for 72 h.

Postoperative opioid analgesia consisted of the administration of morphine 20 mg per day and ketorolac 1 mg per kg per day for 72 h. Administration took place intravenously through an elastomeric system.

### 2.5. Pain Treatment after 72 h

Seventy-two hours after the surgical procedure, patients underwent pain treatment only “as needed”. Therapy consisted in the administration of an oral dose of paracetamol 1 g with the possible addition of oral ketorolac 30 mg in drops. The recommended daily dosage did not exceed 3 g of paracetamol and 60 mg of ketorolac. In patients who showed painful symptoms of NRS greater than 4 beyond 10 days from the surgical procedure, a treatment scheme was set up including the use of paracetamol (up to 3 g per day per os) and opioids (tramadol 100/150 mg per day orally). For patients who began to experience signs of mixed pain, not only nociceptive but also with neuropathic components, pregabalin (up to 300 mg per day orally) or gabapentin (up to 900 mg daily orally) were added. For patients who reported a further increase in the NRS value (greater than 5), tramadol was replaced with oxycodone (up to 30 mg per day orally), or transdermal fentanyl (up to 50 g/h) was added, always in combination with paracetamol and adjuvant drugs (pregabalin or gabapentin).

### 2.6. Early Mobilization Program

Immediately after surgery, patients were encouraged to mobilize with movements of the upper and lower limbs, coordinated with the respiratory acts; as soon as was possible, each patient was encouraged to gradually increase the rhythm and number of movements with the assistance of a healthcare personnel.

### 2.7. Serologic Tests

Hematologic blood tests were performed by routine methods. Serum HBsAg, total anti-HBc, anti-HBs, anti-HCV, and anti-HIV were detected by commercial immunoenzymatic assays as described in previous studies [51,52,53,54,55,56,57,58].

### 2.8. Statistical Analysis

The Wilcoxon signed rank test was used to evaluate whether NRS and MPQ values recorded in individual patients 72 h after biopsy and 6 months later differ significantly (*p* < 0.05). The Yates’ corrected chi-square test was used to assess whether NRS observed within the 72nd hour after biopsy could influence the onset of chronic chest pain, as recorded 6 months later.

## 3. Results

Of the 51 patients included in this study, 17 patients underwent open biopsy via mediastinotomy and 34 by VATS. Early mobilization was performed by all but one patient who did not comply with the early mobilization program due to poor physical condition, intense symptoms, and slow recovery. Forty-six patients were re-evaluated at the 6-month checkpoint, while 6 were lost to follow-up due to lack of cooperation and no longer considered in subsequent evaluations.

The pain evaluated within the 72nd hour after the surgical procedure was very intense (NRS: 10) for one patient only, nine patients had NRS 8, twenty-five NRS 6, and ten NRS 1. The patient with NRS 10 had undergone VATS and was treated with systemic opioid analgesia during hospitalization. Of the nine patients with NRS 8, seven underwent VATS and two mediastinotomy; seven were treated with systemic opioids and two with PVB (Table 1 and Table 2). Of the twenty-five patients with NRS 6, eleven underwent mediastinotomy and fourteen VATS; sixteen were treated with systemic opioids, five with PVB and four with TEA (Table 1 and Table 2). All ten patients with NRS 1 had undergone VATS, seven were treated with systemic opioids, two with PVB and one with TEA (Table 1 and Table 2).

Of the 31 patients who were treated with systemic opioid analgesia after biopsy, NRS values detected at the 6-month check point were significantly lower than those detected within the 72nd hour after biopsy in 29 (Z = −4.778, *p* = 0.001), with a large effect (r = 0.60); in the remaining two the initial NRS score of 6, remained unchanged. MPQ values were also significantly decreased (Z = −4.376, *p* = 0.001) with a large effect (r = 0.55) (Table 1).

For the 5 patients treated with TEA, the NRS values significantly decreased at the 6-month check point (Z = −2.023, *p* = 0.043), with a large effect (r = 0.64). The MPQ values were also significantly decreased at the 6-month checkpoint (Z = −2.032, *p* = 0.042), with large effect (r = 0.64) (Table 2).

For the 9 patients treated with PVB, the NRS values significantly decreased at the 6 month-check point (Z = −2.530, *p* = 0.011), with a large effect (r = 0.59); this score decreased in 8 and persisted with the score 1 in the remaining one. The MPQ values also significantly decreased (Z = −2.668, *p* = 0.008), with a great effect (r = 0.63) (Table 2).

Of the 31 opioid-treated patients re-evaluated at the 6-month checking point, chronic pain was recorded in 8 of 15 with NRS > 6 and in none of 16 with NRS < 6 (*p* < 0.01). At this checkpoint, eight of the 10 patients who underwent mediastinotomy and received opioids had an NRS no greater than 2 and six of these eight had no chronic pain (Table 1). These data were similar for the 21 patients biopsied with VATS and treated with opioids: 15 with NRS no greater than 2, of whom 10 had no chronic pain. A clinically significant chronic pain (NRS > 3) was observed in 2 (20%) of those who underwent mediastinotomy and in 6 (29%) of those biopsied under VATS (Table 1).

**Figure 1:**
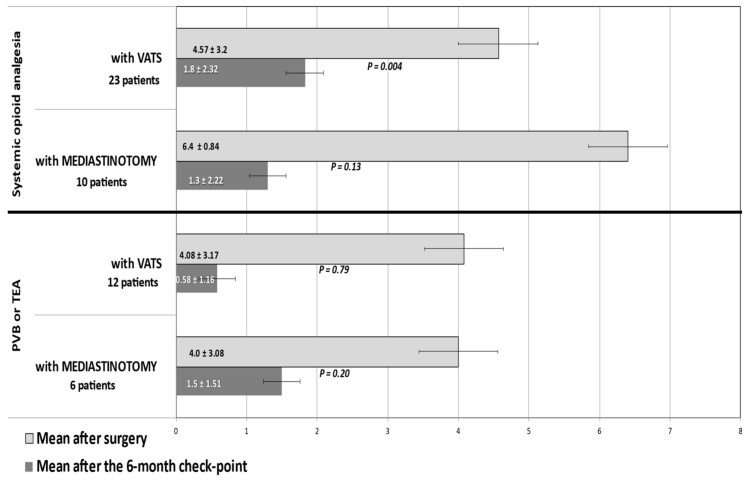
Numerical Rating Scale (NRS) values range 1–10 (mean ± SD) detected after surgery (mediastinotomy or VATS) and after at the 6-month checkpoint in patients treated with opioid analgesia or with TEA or PVB.

The subgroups of patients treated with TEA or PVB, biopsied either under mediastinotomy or VATS, are small, but even in these, an NRS of > 3 at the 6-month checking point was infrequent (Table 2, Figure 1).

Changes in quality of life were consistent with changes in pain intensity in all types of analgesia used (Supplementary data in Tables S1 and S2).

In a multi-logistic analysis, we assessed the age, sex, and pain entity (NRS) after the surgical procedure as independent variables and VATS and mediastinotomy as dependent variables. Although not statistically significant, the odds ratio 0.77 (95% CI: 0.557–1.081, *p* = 0.13) suggests some pain protection of VATS compared to mediastinotomy (Table 3).

## 4. Discussion and Conclusion

Post-thoracotomy pain syndrome (PTPS) is a complication of thoracic surgery, which, if not treated properly, can give rise to a chronic painful post-thoracotomy syndrome that usually reduces the quality of life, especially in patients with lymphomas. Scar pain, a complex entity of multifactorial origin, also has a significant impact, even in minimally invasive surgery. The prevalence of PTPS is described as extremely variable, ranging from 9% to 80% after mediastinotomy and from 5% to 33% after VATS.

Effective perioperative pain management is crucial. TEA is considered the best postoperative pain therapy after mediastinotomy, but when this technique is contraindicated or fails, alternative therapies or pioneering solutions should be chosen [59,60,61,62,63,64]. PBV can be considered as a first-choice treatment in patients for whom VATS biopsy is indicated, and there is no risk of conversion to mediastinotomy. In these cases, it is recommended to initiate analgesic coverage to block nociceptive afferents prior to surgery; regional analgesia should then be continued in the postoperative period, even for 48–72 h after surgery in the case of intense and prolonged pain.

The systemic administration of opioids, the most common method of providing analgesia for postoperative pain for its intrinsic simplicity, is frequently followed by numerous adverse reactions, such as respiratory depression, sedation, nausea, and vomiting.

It should also be noted that the Italian Group of Thoracoscopic Video Surgery suggests that all patients undergoing trans-thoracic biopsy should receive local anesthetic administration with one of the techniques used as an integral part of the VATS analgesic protocol. VATS is generally associated with a lower number of perioperative complications and a reduction in hospital stays. This makes it preferable in general, regardless of the level of anesthetic risk [65]. Although, there are no studies on the disease recurrence rate, several scientific papers document survival at a distance between VATS and thoracotomy surgery [66,67].

We should not forget that the pain due to thoracic surgery has a multifactorial origin, and therefore, it is not surprising that a less invasive surgical procedure may be followed by intense symptoms and vice versa, independently from the anesthetic techniques used [68]; of this, we have clear evidence in the present study.

The anesthetic techniques for pain control and early mobilization used in the present study were useful for most enrolled patients. Indeed, none of them developed infectious complications or sustained post-operative pain, less than a third developed clinically significant chronic pain, and impaired pulmonary ventilation was observed only in one patient unable to perform early mobilization. The observation that out of 31 opioid-treated patients none of the 16 patients with NRS < 6 by the 72nd hour after biopsy developed chronic chest pain, while 8 of 15 with higher values did, supports the opinion we have acquired in conducting the study that, in cases with more intense pain, it is useful to improve the anesthetic treatment already in the first days after the biopsy.

We admit that our data should be considered preliminary because 51 patients may not be quite enough, given the necessary diversifications in surgical and anesthetic choices. However, we believe that our model of integrated clinical practice between hematologists, surgeons, and anesthetists might be borrowed in clinical practice.

## 5. Limitations of the Study

We admit that the present study has some limitations. In fact, pain is a subjective perception which, even if measured with accredited international scales, remains self-declared. Furthermore, many cofactors are implicated in determining pain intensity and duration as patients age. Our patients were predominantly elderly, occupational activity, level of education, level of pain perception, co-pathologies, and many others already listed in this paper. Another limitation is the small size of the sample studied, which however, for the results obtained, we think it may be sufficient to stimulate the setting up of large multicenter studies.

## Figures and Tables

**Table 1:** Patients treated with systemic opioid analgesia after biopsy.

Age (Years)	Sex	Surgery Type	Pain Relief (NRS: 0–10) after Surgery	Pain Relief (NRS: 0–10) after 6 Months	MPQ (PRI: 0–78) after Surgery	MPQ (PRI: 0–78) after 6 Months
64	F	MEDIASTINOTOMY	8	2	58	20
80	M	MEDIASTINOTOMY	6	0	44	5
80	M	MEDIASTINOTOMY	6	4	40	40
71	M	MEDIASTINOTOMY	6	0	44	6
71	F	MEDIASTINOTOMY	6	0	40	3
61	M	MEDIASTINOTOMY	6	1	40	40
62	M	MEDIASTINOTOMY	6	0	44	6
59	M	MEDIASTINOTOMY	6	0	44	4
59	M	MEDIASTINOTOMY	6	0	42	2
64	F	MEDIASTINOTOMY	8	6	56	56
77	M	VATS	8	2	56	20
58	M	VATS	1	0	7	6
60	M	VATS	8	2	56	16
69	M	VATS	6	6	41	41
84	M	VATS	1	0	10	2
74	F	VATS	6	0	44	8
77	F	VATS	8	6	56	50
70	M	VATS	6	6	9	2
72	F	VATS	N.D.	N.D.	N.D.	N.D.
70	F	VATS	6	0	42	6
80	F	VATS	8	6	54	54
70	M	VATS	N.D.	N.D.	N.D.	N.D.
57	M	VATS	6	2	38	20
69	M	VATS	6	2	42	10
84	M	VATS	1	0	6	4
74	F	VATS	10	4	71	62
70	M	VATS	1	0	6	6
76	M	VATS	1	0	7	4
71	M	VATS	1	0	6	3
59	F	VATS	6	0	43	4
61	F	VATS	6	4	42	40
76	M	VATS	1	0	9	2
65	M	VATS	8	2	54	20

Video-Assisted Thoracoscopic Surgery: VATS, the McGill Pain Questionnaire: MPQ, Pain Rating Index: PRI. Not done: N.D.

**Table 2:** Patients treated with paravertebral block (PVB) or epidural analgesia (TEA) after biopsy.

Age (Years)	Sex	Surgery Type	PVB/TEA	Pain Relief (NRS: 0–10) after Surgery	Pain Relief (NRS: 0–10) after 6 Months	MPQ (PRI: 0–78) after Surgery	MPQ (PRI: 0–78) after 6 Months
68	M	MEDIASTINOTOMY	TEA	6	1	40	2
68	F	MEDIASTINOTOMY	TEA	6	2	42	12
64	M	MEDIASTINOTOMY	TEA	6	4	46	40
57	M	MEDIASTINOTOMY	TEA	N.D.	N.D.	N.D.	N.D.
73	M	MEDIASTINOTOMY	TEA	N.D.	N.D.	N.D.	N.D.
62	F	VATS	TEA	1	0	8	0
60	F	VATS	TEA	6	0	40	2
60	F	MEDIASTINOTOMY	PVB	6	2	44	12
64	F	VATS	PVB	8	0	56	2
57	F	VATS	PVB	N.D.	N.D.	N.D.	N.D.
68	F	VATS	PVB	1	1	8	0
72	M	VATS	PVB	6	0	46	6
80	M	VATS	PVB	6	0	42	2
64	F	VATS	PVB	N.D.	N.D.	N.D.	N.D.
67	F	VATS	PVB	1	0	6	0
62	M	VATS	PVB	6	1	44	6
61	M	VATS	PVB	8	4	55	53
73	M	VATS	PVB	6	1	41	6

Paravertebral block: PVB, Epidural analgesia: TEA, Video-Assisted Thoracoscopic Surgery: VATS, the McGill Pain Questionnaire: MPQ, Pain Rating Index: PRI. Not done: N.D.

**Table 3:** Multi-logistic regression analysis to evaluate the entity of pain after surgery in all 51 patients according to the Numerical Rating Scale (NRS): age, sex, and pain entity (NRS) after the surgery were assessed as independent variables and VATS and mediastinotomy as dependent variables.

Vats vs. Mediastinotomy	OR	(95% CI)	*p-*Value
Pain after surgery	0.776	(0.557–1.081)	0.134
SEX (M vs. F)	0.667	(0.166–2.678)	0.568
AGE	1.050	(0.956–1.153)	0.309

## Data Availability

Not applicable.

## Supplementary Materials

The following are available online at https://www.mdpi.com/article/10.3390/healthcare9050589/s1, Table S1: Patient treated with systemic opioids analgesia after biopsy; Brief Pain Inventory, Table S2: Patient treated with paravertebral block (PVB) or epidural analgesia (TEA) after biopsy, Brief Pain Inventory.

## Abbreviations

Epidural analgesia	TEA
Paravertebral block	PVB
McGill Pain Questionnaire	MPQ
Recording the Numerical Rating Scale	NRS
Post-operative pain	PPP
Video-Assisted Thoracoscopic Surgery	VATS
Pain Rating Index	PRI
