# Supplementary material for: Chronic Chest Pain Control after Trans-Thoracic Biopsy in Mediastinal Lymphomas
Source: Healthcare (Basel). 2021 May 18;9(5):589. doi: 10.3390/healthcare9050589 (PMC8157245; doi:10.3390/healthcare9050589)
Supplement: Supplementary file 1 [file healthcare-09-00589-s001.zip › healthcare-1166014-supplementary.pdf]

Table S1: Patient treated with systemic opioids analgesia after biopsy; Brief Pain Inventory

Age (years)	Sex	Surgery type	Brief Pain Inventory (2d part, 0-11) after surgery	Brief Pain Inventory (2d part, 0-11) after 6 months
64	F	MEDIASTINOTOMY	9	3
80	M	MEDIASTINOTOMY	7	1
80	M	MEDIASTINOTOMY	7	4
71	M	MEDIASTINOTOMY	7	1
71	F	MEDIASTINOTOMY	7	1
61	M	MEDIASTINOTOMY	6	2
62	M	MEDIASTINOTOMY	7	0
59	M	MEDIASTINOTOMY	7	0
59	M	MEDIASTINOTOMY	7	1
64	F	MEDIASTINOTOMY	9	5
77	M	VATS	9	1
58	M	VATS	2	1
60	M	VATS	9	1
69	M	VATS	7	7
84	M	VATS	1	1
74	F	VATS	8	1
77	F	VATS	9	5
70	M	VATS	5	5
72	F	VATS	N.D.	N.D.
70	F	VATS	5	0
80	F	VATS	7	5
70	M	VATS	N.D.	N.D.
57	M	VATS	5	1
69	M	VATS	7	2
84	M	VATS	1	1
74	F	VATS	11	5
70	M	VATS	2	1
76	M	VATS	2	1
71	M	VATS	2	1
59	F	VATS	7	1
61	F	VATS	7	5
76	M	VATS	2	1
65	M	VATS	9	3

Video-Assisted Thoracoscopic Surgery: VATS, the McGill Pain Questionnaire: MPQ, Pain Rating Index: PRI. Not done: N.D.

Table S2: Patient treated with paravertebral block (PVB) or epidural analgesia (TEA) after biopsy, Brief Pain Inventory

**Age (years)**	**Sex**	**Surgery type**	**PVB/ TEA**	**Brief Pain Inventory (2d part, 0-11) After Surgery**	**Brief Pain Inventory (2d part, 0-11) After 6 months**
68	M	MEDIASTINOTOMY	TEA	7	1
68	F	MEDIASTINOTOMY	TEA	7	1
64	M	MEDIASTINOTOMY	TEA	5	3
57	M	MEDIASTINOTOMY	TEA	N.D.	N.D.
73	M	MEDIASTINOTOMY	TEA	N.D.	N.D.
62	F	VATS	TEA	2	1
60	F	VATS	TEA	7	1
60	F	MEDIASTINOTOMY	PVB	6	1
64	F	VATS	PVB	9	1
57	F	VATS	PVB	N.D.	N.D.
68	F	VATS	PVB	2	1
72	M	VATS	PVB	7	1
80	M	VATS	PVB	6	1
64	F	VATS	PVB	N.D.	N.D.
67	F	VATS	PVB	2	1
62	M	VATS	PVB	5	1
61	M	VATS	PVB	7	3
73	M	VATS	PVB	7	1

Paravertebral block: PVB, Epidural analgesia: TEA, Video-Assisted Thoracoscopic Surgery: VATS, the McGill Pain Questionnaire: MPQ, Pain Rating Index: PRI. Not done: N.D.
